# Oleanolic acid induces migration in Mv1Lu and MDA-MB-231 epithelial cells involving EGF receptor and MAP kinases activation

**DOI:** 10.1371/journal.pone.0172574

**Published:** 2017-02-23

**Authors:** Ángel Bernabé-García, David Armero-Barranco, Sergio Liarte, María Ruzafa-Martínez, Antonio Jesús Ramos-Morcillo, Francisco José Nicolás

**Affiliations:** 1 Laboratorio de Oncología Molecular y TGF-ß, Instituto Murciano de Investigaciones Biosanitarias-Arrixaca, Hospital Clínico Universitario Virgen de la Arrixaca, El Palmar, Murcia, Spain; 2 Departamento de Enfermería, Facultad Enfermería, Universidad de Murcia, Murcia, Spain; University of South Alabama Mitchell Cancer Institute, UNITED STATES

## Abstract

During wound healing, skin function is restored by the action of several cell types that undergo differentiation, migration, proliferation and/or apoptosis. These dynamics are tightly regulated by the evolution of the extra cellular matrix (ECM) contents along the process. Pharmacologically active flavonoids have shown to exhibit useful physiological properties interesting in pathological states. Among them, oleanolic acid (OA), a pentacyclic triterpene, shows promising properties over wound healing, as increased cell migration *in vitro* and improved wound resolution *in vivo*. In this paper, we pursued to disclose the molecular mechanisms underlying those effects, by using an *in vitro* scratch assay in two epithelial cell lines of different linage: non-malignant mink lung epithelial cells, Mv1Lu; and human breast cancer cells, MDA-MB-231. In every case, we observed that OA clearly enhanced cell migration for *in vitro* scratch closure. This correlated with the stimulation of molecular pathways related to mitogen-activated protein (MAP) kinases, as ERK1,2 and Jun N-terminal kinase (JNK) 1,2 activation and c-Jun phosphorylation. Moreover, MDA-MB-231 cells treated with OA displayed an altered gene expression profile affecting transcription factor genes (*c-JUN*) as well as proteins involved in migration and ECM dynamics (*PAI1*), in line with the development of an epithelial to mesenchymal transition (EMT) status. Strikingly, upon OA treatment, we observed changes in the epidermal growth factor receptor (EGFR) subcellular localization, while interfering with its signalling completely prevented migration effects. This data provides a physiological framework supporting the notion that lipophilic plant extracts used in traditional medicine, might modulate wound healing processes *in vivo* through its OA contents. The molecular implications of these observations are discussed.

## Introduction

During wound healing, skin function is restored by the action of numerous cell types. These cells undergo proliferation, differentiation, migration, and apoptosis [1]. Regular wound healing is characterized by three overlapping phases: inflammatory, proliferative, and remodelling. In the first phase, the instantaneous response triggers a cascade of events that ends in the formation of a three-dimensional structure, the fibrin clot, that halts bleeding and will serve as provisional matrix for the migration of inflammatory and structural cells to the wound site [2]. Besides, wound healing is a complex process orchestrated by many growth factors and cytokines, which explains the multiple growth factor receptors present in these cells [3]. Among those, IL-1, EGF, or TGF-ß, are known to play important roles [1]. These factors are released by a variety of cells (e.g., platelets, neutrophils, fibroblasts, endothelial cells, macrophages, and lymphocytes) and they accumulate within the provisional matrix and ECM [4].

The most limiting aspect for the wound healing process is cell migration, as defects in this function, but not in proliferation or differentiation, are associated with the clinical phenotype of chronic non-healing wounds [5]. In dermal wounds granulation tissue, platelets, monocytes and other blood cellular constituents release diverse growth factors which stimulate migration of fibroblasts into the wound site, were they proliferate and commit in the reconstitution of connective tissue components [6]. Since wound healing necessitates cell migration, substances promoting cell migration are useful to improve wound repair [7].

Several plant-derived polyphenols are known to promote cell migration, thus improving wound healing [8]. Flavonoids, a sub-group of polyphenols, are major active components in plants which are considered components of food that provide medical or health benefits [9]. They have diversified structures and have been reported to promote pharmacological effects, including wound-healing induction [8]. In that sense, several studies point out to Oleanolic Acid (OA), a pentacyclic triterpenoid compound with a widespread occurrence throughout the plant kingdom, as the main component of natural plant extracts with wound healing properties [10]. For instance, OA application on wounds of mice increases their tensile strength [11]. Moreover, while its use in human wounds as part of a mixture of components rendered an accelerated healing process and better aesthetic results [12], also showing apparent ulcer-healing properties [13]. Not surprisingly, application of OA has been shown to increase migration of NIH-3T3 cells in a wound healing scratch assay [14]. However, despite the accumulated evidence, little research has been done on the cellular mechanisms driving those effects.

In this paper, we tried to address the molecular events driven by OA treatment towards enhanced cell motility using reference cell lines to study migration. We demonstrate that OA potent pro-migratory effects depend on the activation of several factors critical for cell migration, been mainly driven through mitogen-activated protein (MAP) kinases. Moreover, and despite its independence from cell proliferation, we demonstrate the up-stream involvement of the EGF-receptor (EGFR) in this process. Finally, we show how OA treatment is able to alter the expression profile of key genes involved in the migratory status and tissue remodelling capabilities of cells.

## Materials and methods

### Preparation of oleanolic acid

Oleanolic acid (OA, purity > 98%) (Nutrafur-Frutarom Group, Murcia, Spain), was usually dissolved in DMSO to a 1:1000 ratio to the final assay concentration. DMSO presence in all cell culture assays never surpassed the 1% concentration. OA concentration used is indicated at each experiment.

### Cell culture and wound healing scratch assay

Mink Lung Epithelial (Mv1Lu) [15–17] cells were grown in Eagle’s Minimum Essential Medium (EMEM) (Biowest, Nuaillé, France). Human Mammary Gland cells (MDA-MB-231) [18–20] were grown in Dulbecco’s Modified Eagle Medium (DMEM) (Biowest, Nuaillé, France). Both media were supplemented with 10% Fetal Bovine Serum (FBS), 1% Penicillin/Streptomycin and 1% L-Glutamine (all from Biowest, Nuaillé, France). Cells were incubated in a humidified atmosphere at 37°C with 7.5% CO_2_.

For wound healing scratch assay, 95% confluent cells were detached by 0.05% trypsin/EDTA (Sigma-Aldrich, St Louis, MO, USA) and seeded for cell expansion on either 50 mm diameter plates. Cells were grown for five days, then growth medium was changed for complete DMEM deprived of FBS (0% FBS) for MDA-MB-231 cells or complete EMEM deprived of FBS (0% FBS) for Mv1Lu cells for 24 hours before wound healing scratch assay. When indicated, immediately before wounding, cells were treated for 3 h with 10 μg/ml of Mitomycin-C (MMC) (Sigma-Aldrich, St Louis, MO, USA) to prevent cell proliferation. Wound was made by scratching a line across the bottom of the dish on a confluent cells monolayer using a sterile p-200 pipette tip. Cells were rinsed very gently with phosphate buffered saline (PBS) (Biowest, Nuaillé, France) and then the cells were cultivated in the corresponding medium deprived of serum supplemented with either Epidermal Growth Factor (EGF) 10 ng/ml (Sigma-Aldrich, St Louis, MO, USA) or OA when corresponding. The following pharmacological inhibitors were added to the medium where indicated: JNK inhibitor SP600125 (JNKi), 15 μM; MEK inhibitor PD98059 (MEKi), 50 μM; EGFR inhibitor PD153035 (EGFRi), 10 μM (all from Sigma-Aldrich, St Louis, MO, USA). Also where indicated, immediately after wounding, cells were treated with 0.2 μg/ml of anti-EGFR blocking antibody 5 min prior to stimulus. Pictures were taken at 10x magnification using an optical microscope equipped with a digital camera (Motic Optic AE31, Motic Spain, Barcelona, Spain). To quantify migration of the wound healing scratch assay, the area of the gap obtained by scratching a line across the bottom of the dish was quantified. Upon completion of the assay, the area of the same gap was measured again and differences between initial and final images were calculated and expressed as percentage of covered area. In every case, image processing to obtain quantitative data was accomplished by using *Image-J* software (http://rsbweb.nih.gov/ij/). We interpreted bigger differences as a reflection of better migration. That difference was represented for each treatment.

### Proliferation and cell cycle assays

Long-term cell proliferation was evaluated by cell counting. Briefly, cells were seeded in a 50-mm dish at a density of 10^5^ cells/plate in complete medium supplemented with 10%FBS and treated in duplicates with 75μM or 100μM OA depending on the cell line (Mv1Lu or MDA-MB-231, respectively). At the indicated days, control and treated cells were detached using trypsin/EDTA (Biowest, Nuaillé, France) and harvested. Cell count was performed using a TC10 automated cell counter (Bio-Rad, Hercules, Ca, USA) and viability of cells was determined by trypan blue dye (Sigma-Aldrich, St Louis, MO, USA) exclusion assay. Data were plotted as a growth curve. For cell cycle analysis, samples from detached cells were taken and centrifuged and the pellet of cells was immediately fixed with ice cold 70% ethanol. Cells were then washed three times with cold PBS (Biowest, Nuaillé, France) to remove ethanol and finally stained for cell cycle with propidium iodide using a routine method. Briefly, cells were treated with a solution of 20 μg/ml Ribonuclease A (Sigma-Aldrich, St Louis, MO, USA) and 40 μg/ml propidium iodide (Sigma-Aldrich, St Louis, MO, USA) in PBS. Then, cells were analysed by flow cytometry using a FACS Calibur 1 (Beckton Dickinson, Franklin Lakes, NJ, USA).

For short-term cell proliferation evaluation, cells were formalin fixed 24 h after treatments and immunostained for phospho-Histone3 (Santa Cruz Biotechnology, Santa Cruz, CA). After labeling with the primary antibody, samples were stained with the appropriate fluorescent-labelled secondary antibodies together with Hoechst 33258 (Fluka, Biochemika, Sigma-Aldrich, St Louis, MO, USA) (1.2 μg/ml) for 1 hour at room temperature. Ten field images were taken randomly with a confocal microscope (LSM 510 META from ZEISS, Jena, Germany). Pictures were examined and either positive or negative nuclei were counted in order to determine percentage of positive nuclei per field.

### Western blot

Sub-confluent cells (50%) serum-deprived for 24 hours were treated with either EGF or OA and in the presence or absence of pharmacological inhibitors for the indicated times. When indicated, cells were treated with ribosomal inhibitor Geneticin (G418) [21] at 1 mg/ml 30 minutes before the main stimulation to prevent de novo synthesis of proteins. Cells were harvested and lysed with 20 mM TRIS pH 7.5, 150 mM NaCl, 1 mM EDTA, 1.2 mM MgCl2, 0.5% Nonidet p40, 1 mM DTT, 25 mM NaF and 25 mM beta-glycerophosphate supplemented with phosphatase inhibitors (I and II) and protease inhibitors (all from Sigma-Aldrich, St Louis, MO, USA). The extracts were analysed by SDS-PAGE followed by Western blot (WB) using the appropriate antibodies. WBs were developed using Horseradish peroxidase substrate (ECL) (GE Healthcare, GE, Little Chalfont, United Kingdom) and images were taken with a Chemidoc XRS® (Bio-Rad, Hercules, Ca, USA).

### Immunocytochemistry

Sub-confluent cells (50%) serum-deprived for 24 hours were treated with either EGF or OA and in the presence or absence of pharmacological inhibitors for the indicated times. After indicated periods, cells were processed as described elsewhere [22]. Briefly cells were fixed with 4% formaldehyde and subsequently permeabilized in a 0.3% Triton X-100 solution, and then exposed to blocking buffer. Samples were incubated for 2 h with anti-EGFR antibody diluted in blocking buffer. Appropriate fluorescent-labelled secondary antibody was incubated together with Alexa fluor594-labelled phalloidin (Molecular Probes, Thermo Fisher Scientific, Waltham, MA USA) and Hoechst 33258 (Fluka, Biochemika, Sigma-Aldrich, St Louis, MO, USA) for 1 h at room temperature. Samples were examined and representative images were taken with a confocal microscope (LSM 510 META from ZEISS, Jena, Germany).

### Antibodies

The following commercial antibodies were used: anti-ERK1/2, anti-phospho-ERK1/2, anti-JNK1/2, anti-phospho-JNK1/2, and anti-phospho-c-Jun (all Cell Signaling Technology, Danvers, MA, USA); anti-c-Jun, anti-phospho-Histone3, anti-p21 and anti-EGFR (Santa Cruz Biotechnology, Heidelberg, Germany); anti-phospho-EGFR and blocking anti-EGFR (Thermo Fisher Scientific, Rockford, IL, USA); anti-Paxillin (Abcam, Cambridge, MA, USA); and anti-β-Actin (Sigma-Aldrich, St Louis, MO, USA). Secondary antibodies were anti-rabbit IgG Horseradish peroxidase linked F(ab’)_2_ I fragment (from donkey) (GE Healthcare, GE, Little Chalfont, United Kingdom), Horseradish peroxidase linked Rat anti-mouse IgG_1_ (BD Pharmingen, Beckton Dickinson, Franklin Lakes, NJ, USA) and Alexa Fluor 488 conjugated anti-mouse IgG (from donkey) (Thermo Fisher Scientific, Rockford, IL, USA).

### Statistics

Data of the wound healing assays were analysed with analysis of variance (ANOVA) using the using Prism’s Graph Pad Data Analysis software. *P*-values lower than 0.05 were considered to be statistically significant. Gene expression and proliferation data were analysed by unpaired two-tailed Student’s t-test to determine differences between the samples using Prism’s Graph Pad Data Analysis software. At the figure legends, the asterisks denote statistically significant differences between the treatments (*p<0.05, **p<0.005, ***p<0.001 and ****p<0.0001).

### Quantitative PCR

Sub-confluent cells (50%) serum-deprived for 24 hours were treated with OA for 6 and 24 hours and RNA was extracted using the RNeasy-mini system (Qiagen, Venlo, The Nederlands). Typically, 1 μg of RNA from independent samples was retro-transcribed using iScript reagents (Bio-Rad, Hercules, CA, USA), and resulting cDNA was used for quantitative PCR (qPCR) using the SYBR premix ex Taq kit (Takara Bio Europe/Clontech, Saint-Germain-en-Laye, France) according to the manufacturer’s instructions. For each mRNA, gene expression levels were normalized to Glyceraldehyde 3-phosphate dehydrogenase (GAPDH) content of each sample by applying the comparative Cq method (2^-∆∆Cq^). Two samples of three independent experiments were quantified by qPCR, analysed data represent mean ± SEM. Primers used for gene amplification are detailed in Table 1.

**Table 1:** Primers used for quantitative PCR.

Sequence 5’ to 3’	Name of the primer
GGAAACGACCTTCTATGACGATGCCC	c-JUN-F
GGCGCGCACGAAGCCCTCGGCGAACC	c-JUN-R
CATGGGGCCATGGAACAAGG	PAI1-F
CTTCCTGAGGTCGACTTCAG	PAI1-R
GATGCCGCGCTCCTTCCTGGTC	SNAI2-F
GCTGCTTATGTTTGGCCAGCC	SNAI2-R
ATGTCAGAACCGGCTGGGGATG	CDKN1A-F
GGGCTTCCTCTTGGAGAAGATC	CDKN1A-R
CTCCGCCTCCATGGATGACG	ITGB6-F
CCAAGACAGTTGACATGGAG	ITGB6-R
ACCACAGTCCATGCCATCAC	GAPDH-F
TCCACCACCCTGTTGCTGTA	GAPDH-R
Proprietary sequence (Qiagen QuatiTect)	COL1A1
Proprietary sequence (Sigma Quick Start)	EGF
Proprietary sequence (Qiagen QuatiTect)	FOXO1
Proprietary sequence (Qiagen QuatiTect)	MMP9
Proprietary sequence (Qiagen QuatiTect)	PAX

## Results

### Oleanolic acid stimulates cell migration in Mv1Lu cells

**Fig 1:**
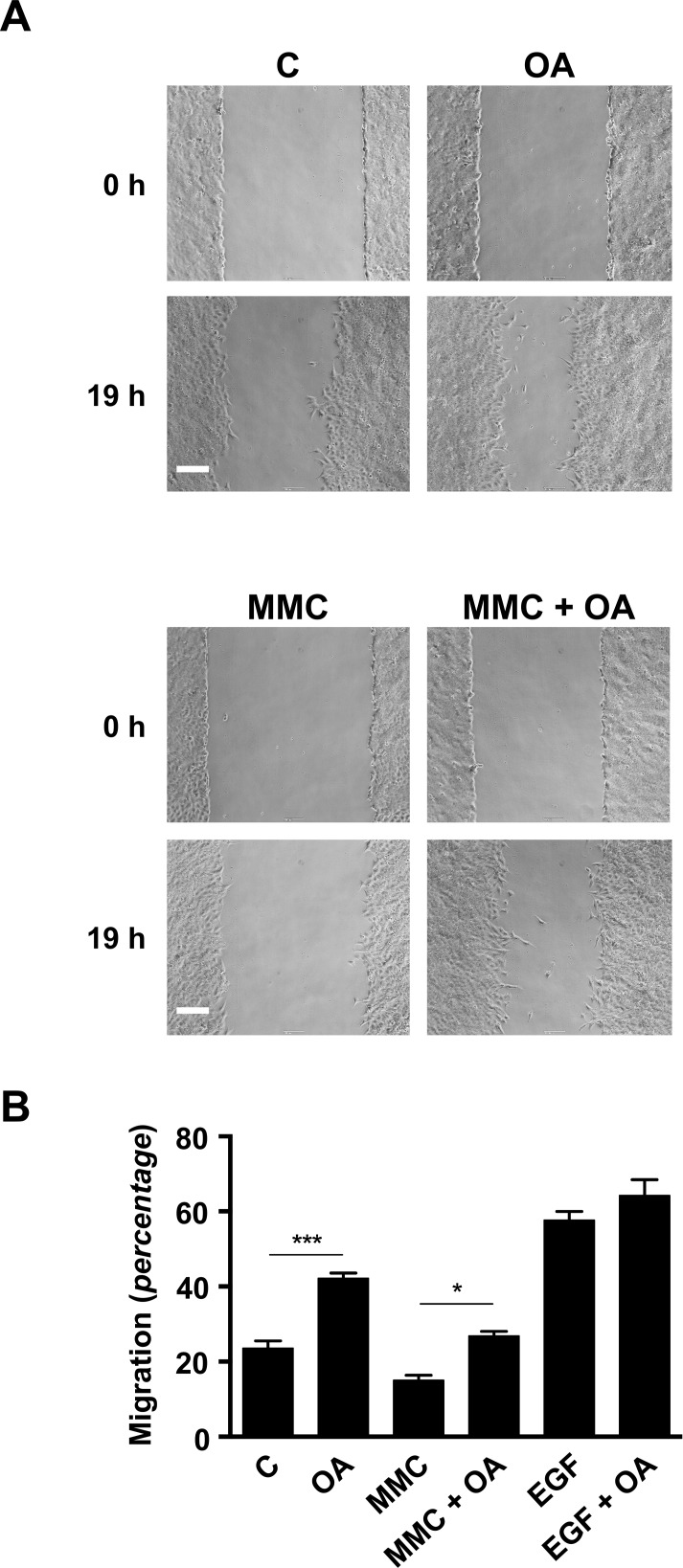
Oleanolic acid enhances migration in Mv1Lu cells. (A) The figure shows micrographs of the extent of closure obtained under control conditions compared to those with OA after 19 h treatment in serum-free medium. A second batch of samples was pre-treated with Mytomicin-C (MMC) to discriminate proliferation’s contribution to Mv1Lu migration. Phase-contrast microscopy pictures of the wounded area were taken. Representative pictures are shown. Scale Bar 200 μm. (B) Cell migration is represented as variation of the area differences between treatments and control samples for each assay (percentage). The plot is representative of three independent experiments. *p<0.05, **p<0.005, ***p<0.001 and ****p<0.0001.

In this work we have explored the underlying molecular mechanisms responsible for the effects of OA over the wound healing process. Non-malignant mink lung epithelial cells Mv1Lu, which are recognized as a good model for cell migration studies [15–17], were used in wound healing scratch experiments to assess OA properties. A confluent culture monolayer of Mv1Lu cells was scratched and OA was added to the medium. Several concentrations of OA were empirically tested to optimize the assay (S1A and S1B Fig). Interestingly, high doses of OA were necessary to obtain marginal increases in migration (S1A Fig) when serum supplemented medium was used, while relatively low doses were capable to clearly enhance migration in serum deprived conditions, finding 5μM OA to yield the best performance (S1B Fig). As recorded in phase-contrast microscopy pictures (Fig 1A), treatment with 5 μM OA significantly induces migration of Mv1Lu cells into the scratch area when compared to untreated cells. When the different migration behaviours were analysed by means of imaging software, OA stimulation was found to consistently enhance Mv1Lu cells migration when compared to basal conditions (Fig 1B). However, OA treatment showed no additive effect on the cell migration behaviour triggered upon EGF treatment positive control (Fig 1B). Strikingly, when proliferation was chemically disrupted due to the application of Mitomycin-C (MMC) to the medium, OA stimulation on migration remained noticeable (Fig 1A and 1B).

Altogether, these results evidence that OA treatment is able to induce Mv1Lu cells migration in a process that, at least in part, would be independent from cell proliferation.

### Oleanolic acid induces ERK and JNK signalling pathways

**Fig 2:**
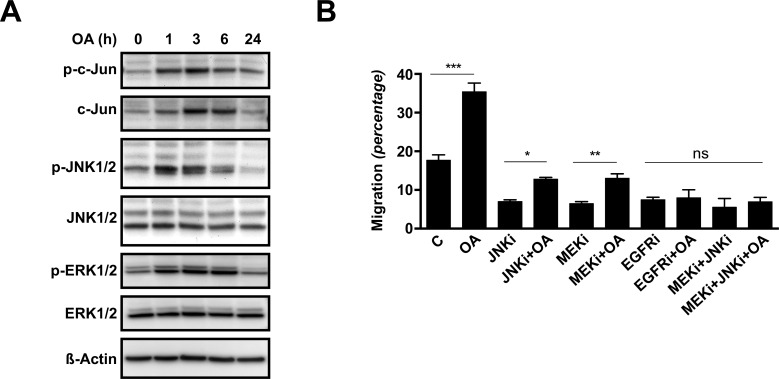
Oleanolic acid triggers phosphorylation of c-Jun, ERK1/2 and JNK1/2 kinases, while the inhibition of this activation prevents pro-migratory effects in Mv1Lu cells. (A) Total cell lysates from serum-deprived, sub-confluent Mv1Lu cells treated with 5μM Oleanolic Acid (OA) for different times were analyzed by Western blot for phospho-c-Jun, c-Jun, phospho-ERK1/2 and phospho-JNK1/2 proteins. ß-Actin was used as a protein loading control. Lines isolating initial times indicate that distant areas of the same blot were fused together. A representative image of at least three independent experiment is shown. (B) Wound healing scratch assay was performed on confluent Mv1Lu cells and cell migration quantification was determined (percentage). Cells were treated either with 15 μM SP600125 (JNKi), 50 μM PD98059 (MEKi) or 10 μM PD153035 (EGFRi), or combinations of them; in the presence or absence of 5 μM OA in serum-free medium. The plot is representative of three independent experiments. Note that JNK inhibitor and MEK inhibitors are capable to interfere with OA, while either EGF-Receptor inhibitor alone or combination of JNK and MEK inhibitors hampered any observable OA effects. *p<0.05, **p<0.005, ***p<0.001 and ****p<0.0001.

It is known that MAP kinases such as ERK and JNK are key players in the regulation of cell migration. To study whether these signalling pathways participate in the observed stimulatory effect of OA over Mv1Lu cells migration, we analysed changes in protein levels and protein-phosphorylation of total extracts from sub-confluent Mv1Lu cells stimulated for various periods of time with 5μM OA. Upon treatment, phosphorylated-c-Jun levels increased and remained elevated after 6 h of exposure. Simultaneously, total c-Jun protein levels registered a steady increase along the 24 h treatment (Fig 2A). Consistently, treatment of Mv1Lu cells with OA sharply induced phosphorylation of JNK1/2 kinases, which faded after 6 h treatment (Fig 2A). However, neither phosphorylation nor induction of the MAP kinase p38 was observed (data not shown). Finally, treatment with OA also induced the phosphorylation of ERK1/2 kinases, which portrayed a distinct time dependent pattern (Fig 2A).

In sum, the results show that OA can activate ERK and JNK signalling pathways and induce the up-regulation and phosphorylation of c-Jun.

### MEK, JNK and EGFR inhibitors prevent oleanolic acid-induced cell migration

We have shown that OA stimulates c-Jun phosphorylation and up-regulates its protein levels. In order to discriminate the events leading to these effects, we performed a wound healing scratch assay on Mv1Lu cells in the presence of OA along with established inhibitors of various signalling pathways involved in cell migration: p38 inhibitor SB203580, MEK inhibitor PD98059 (MEKi), JNK inhibitor SP600125 (JNKi) and EGFR inhibitor PD153035 (EGFRi). After 24 hours, the wounded area was examined by phase-contrast microscopy (S2 Fig). Consistently with WB observations, p38 inhibition did not have any significant effects on OA stimulation of Mv1Lu cell migration (data not shown). However, this stimulation was partially prevented by MEKi and JNKi, while treatment with EGFRi completely abrogated the OA effects (Fig 2B and S2 Fig). As MEK and JNK signalling are both located downstream the EGFR, we performed additional assays on Mv1Lu cells exposed to combinations of the aforementioned inhibitors. Indeed, cells exposed to OA in combination with MEKi plus JNKi achieved similar migratory performance than cells exposed to EGFRi or EGFRi and OA (Fig 2B).

Everything included, these results strongly suggest that OA induces cell migration with the involvement of active MEK and JNK kinases signalling under the influence of the EGFR activities.

### Oleanolic acid properties on cell proliferation are detached from Mv1Lu migration

**Fig 3:**
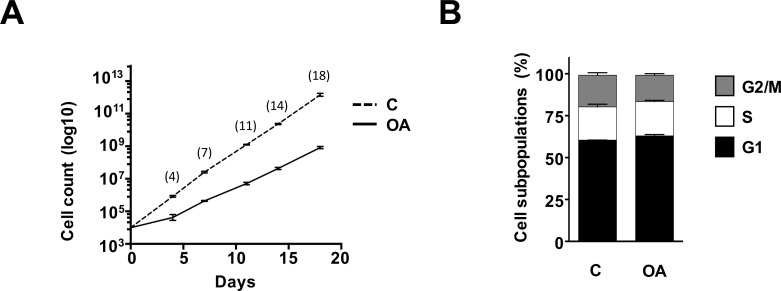
Oleanolic acid effects on Mv1Lu proliferation do not partake for migration performance on serum depleted conditions. (A) Long term effects of OA on Mv1Lu proliferation was assessed by counting total cells at the indicated times. The logarithm of the mean number of cells against time is plotted. (B) Cell cycle subpopulations composition was evaluated on cells obtained from long term culture passages. All plots are representative of at least three independent experiments.

As previously shown in Fig 1, the positive effects of OA over Mv1Lu cell migration prevailed despite the application of MMC. Strikingly, OA also triggered c-Jun phosphorylation (Fig 2A), which on top of its migratory significance it also has implications on the proliferative status [23–25]. These observations prompted us to study more in depth the effects of OA on the Mv1Lu mitogenic activity. Long term culture of Mv1Lu cells using serum-supplemented medium containing 75μM OA revealed mild anti-proliferative effects for this flavonoid when compared to controls (Fig 3A), similarly to previous reports [14, 26]. Further analysis of the effects of OA over Mv1Lu cell cycle using the same conditions, revealed no apparent alterations among the different cellular subpopulations of OA treated Mv1Lu cells (Fig 3B). This led us to study shorter-term effects of OA on proliferation. For that purpose, we studied the detection of phosphorylated-Histone H3 (PHH3) using specific antibodies on 24 h OA exposed formalin fixed cells. PHH3 reaches a maximum during mitosis, therefore it can be used as a specific mitotic marker [27]. In line with previous observations, immune-fluorescence analysis performed on OA treated samples cultured in serum-supplemented conditions containing 75 μM OA, reported clear reduction in mitotic activity compared to controls (S3A and S3B Fig). However, when PHH3 immune-detection was studied in serum deprived cultures, no significant differences could be found between cells exposed to 5 μM OA and controls (S3C and S3D Fig). These findings were also corroborated in Mv1Lu confluent monolayers that underwent scratching procedure (data not shown).

As a whole, these observations support the notion that the effects of OA treatment to Mv1Lu cells, although partially contributed by cell proliferation, would involve an independent migration component.

### Oleanolic acid stimulates cell migration involving erk and jnk signalling pathways in human-derived MDA-MB-231 cells

As epithelial cells are directly involved in the re-epithelialization phase of the altered external lining in the wound healing process, we used a breast cancer epithelial cell line: MDA-MB-231, a suitable human model to asses OA effects on cell migration [18–20]. Due to its tumour origin MDA-MB-231 display some interesting properties for our studies, as high endogenous migratory capacities and the ability to sustain certain degree of proliferation under serum deprived culture conditions.

**Fig 4:**
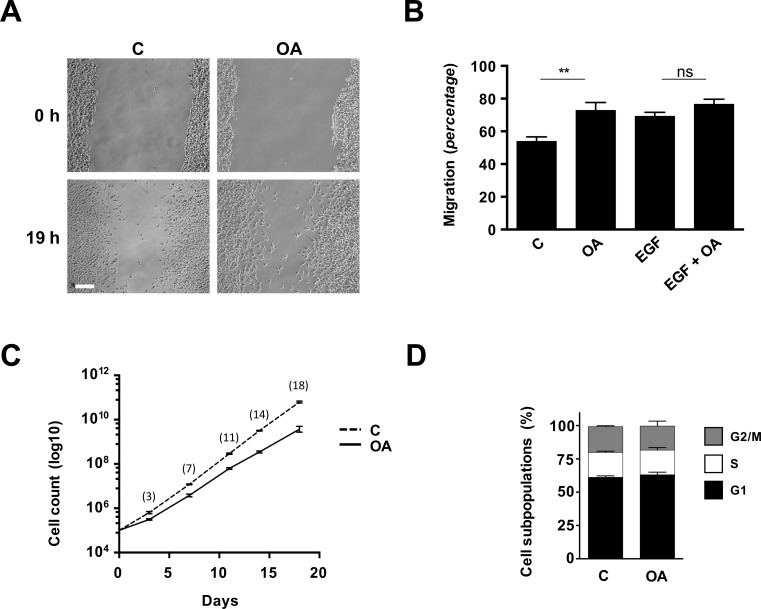
Oleanolic acid stimulates scratch closure in MDA-MB-231 cells independently of proliferation. (A) The figure shows micrographs of the extent of closure obtained under control conditions compared to those with OA (10 μM) after 19 h treatment. A representative picture is shown. Scale Bar 200 μm. (B) Cell migration quantification was assessed by determining differences in the central gap area (see Materials and Methods). Cell migration is represented as variation of the area differences between treatments and control samples for each assay (percentage). (C) Long term effects of OA on proliferation were assessed by counting total cells at the indicated times. The logarithm of the mean number of cells against time is plotted. (D) Cell cycle subpopulations composition was evaluated on cells obtained from long term culture passages. All plots are representative of at least three independent experiments. *p<0.05, **p<0.005, ***p<0.001 and ****p<0.0001.

Upon OA treatment, MDA-MB-231 cells developed a similar migratory response to OA to that of Mv1Lu cells (Fig 4A and 4B), even though these cells seem to tolerate OA presence to a greater extent than Mv1Lu cells and show less apparent differences in migration between control and OA induced conditions in both serum deprived and serum supplemented circumstances (S3A and S3B Fig). Also similarly to Mv1Lu, long term (72 h) MDA-MB-231 cultures exposed to OA shown a mild reduction of cumulative cell proliferation, which is not capable to produce any significant variation on the subcellular population composition (Fig 4C and 4D). Interestingly, we observed how cultures of MDA-MB-231 cells treated with OA under both serum deprived and supplemented conditions displayed a slight yet significant reduction in phospho-histone 3 immune-labelling (S4C, S4D, S4E and S4F Fig), effect which may contribute to the observed reduction in the cumulative proliferation. This, along with the fact that no relevant increase in migration was recorded on OA-EGF co-treated MDA-MB-231 cells (data not shown), further support the impression that the observed contribution of OA to migration of MDA-MB-231 cells is independent of cell proliferation.

**Fig 5:**
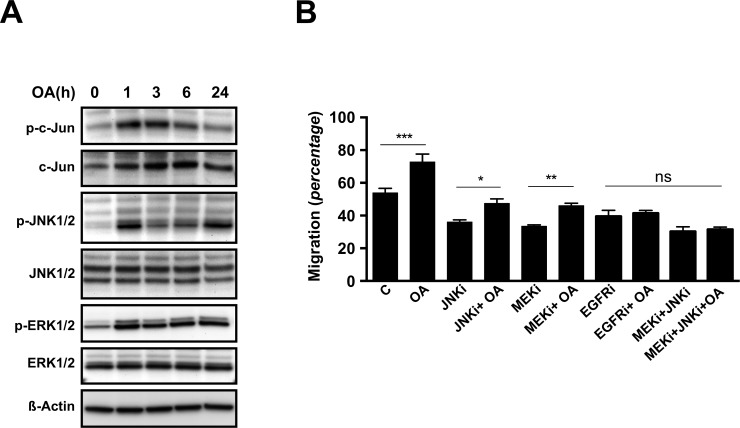
Oleanolic acid triggers phosphorylation of c-Jun, ERK1/2 and JNK1/2 kinases, while the inhibition of this activation prevents pro-migratory effects in MDA-MB-231 cells. (A) Total cell lysates from serum-deprived, sub-confluent MDA-MB-231 cells treated with 10μM Oleanolic Acid (OA) for different times were analyzed by Western blot for phospho-c-Jun, c-Jun, phospho-ERK1/2 and phospho-JNK1/2 proteins. ß-Actin was used as a protein loading control. Lines isolating initial times indicate that distant areas of the same blot were fused together. A representative image of at least three independent experiment is shown. (B) Wound healing scratch assay was performed on confluent MDA-MB-231 cells and cell migration quantification was determined (percentage). Cells were treated either with 15 μM SP600125 (JNKi), 50 μM PD98059 (MEKi), 10 μM PD153035 (EGFRi) or combinations of them; in the presence or absence of 10 μM OA in serum-free medium. The plot is representative of three independent experiments. Note that JNK inhibitor and MEK inhibitors are capable to interfere with OA, while EGF-Receptor inhibitor alone or combination of JNK and MEK inhibitors hampered any observable OA effects. *p<0.05, **p<0.005, ***p<0.001 and ****p<0.0001.

To corroborate the earlier observations made on the OA effects on the MAPK signalling pathways, we applied 10μM OA treatments over sub-confluent MDA-MB-231 cells and obtained total protein extracts over the time. WB analysis showed that in MBA-MB-231 cells OA induces phosphorylated-c-Jun levels clearly after 1 h, peaking at 3 h time to steadily decrease towards control levels after 24 h (Fig 5A). In a similar fashion, c-Jun protein levels increased markedly at short time and dropped back 24 h after treatment (Fig 5A). The OA treatment was also capable to induce phosphorylation of JNK1/2 kinases in MDA-MB-231, but unlike in Mv1Lu cells, this induction of phosphorylation remained conspicuous over time (Fig 5A). Moreover, MDA-MB-231 cells experienced initially a sharp increase in the phosphorylation of ERK1/2 kinases, which diminished mildly overtime (Fig 5A). Consequently, these data show that in the human epithelial model MDA-MB-231, OA is also capable of triggering ERK and JNK signalling pathways, while inducing the up-regulation and phosphorylation of c-Jun.

To further validate the involvement of the previously underlined molecular pathways in the migration of human cells, inhibitors treatment along with OA was applied on MDA-MB-231 cells in a scratch assay. Anew, MDA-MB-231 depicted a similar behaviour to that of Mv1Lu cells (S5 Fig), defined by partial inhibition of OA’s induction of migration achieved by JNKi and MEKi separate treatments, while treatment with EGFRi alone abolished any sign of OA’s effects (Fig 5B). Also in this case, concurrent treatment with JNKi plus MEKi equalized the EGFRi and the EGFRi plus OA treatments behaviour (Fig 5B).

Utterly, these results certify the ability of OA to induce cell migration through MEK and JNK MAP kinases signalling pathways, involving the upstream activity of EGFR. Moreover, these observations provide solid evidence of the OA properties on cell migration in a convenient human cell migration model.

### Oleanolic acid alters the expression profile of genes involved in cell migration

**Fig 6:**
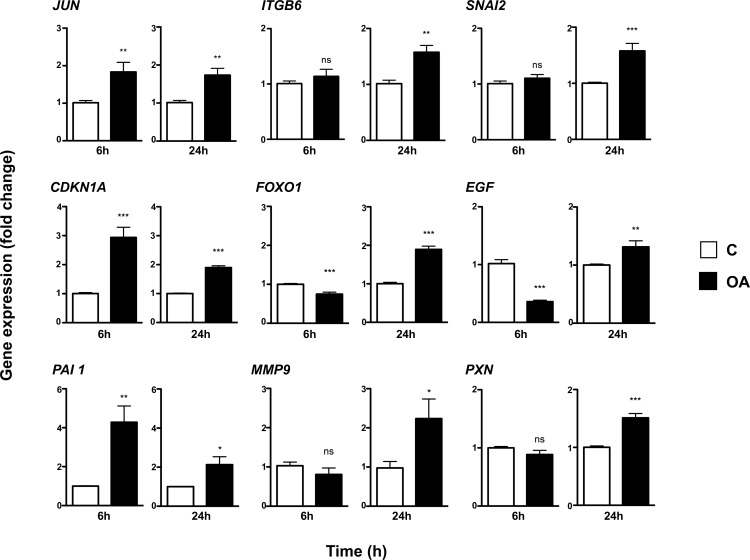
Oleanolic acid induces the expression of genes involved in cell migration and proliferation in MDA-MB-231 cells. MDA-MB-231 cells were treated with 10μM Oleanolic Acid (OA) for 6 and 24 hours. Expression levels data is represented as fold change from control samples. Shown data is average of three independent experiments. *p<0.05, **p<0.005, ***p<0.001 and ****p<0.0001.

To better understand the stimulatory effect of OA on cell migration modulation and proliferation, we studied its effect on the transcription of a subset of genes known to be involved in those processes (Fig 6). For this purpose, we used MDA-MB-231 samples, as availability of Mink sequences and primer sets is highly limited.

As it was expectable, Real-time PCR analyses showed that MDA-MB-231 cells exhibit clear induction of the *c-JUN* gene at 6 h after OA treatment begins, with increased levels remaining elevated at 24 h as well. It has been shown that JNK leads to the induction of downstream target genes, e.g. Plasminogen Activator Inhibitor 1 (*PA-1*) [28], which protein product has been shown to be crucial for migration, in addition to its role in proteolysis control [29]. Noticeably, the transcription levels of *PAI-1* showed a marked response upon OA stimulation at 6 h, partially sustaining this increase over the 24 h period. Likewise, the *CDKN1A* gene, responsible for the cyclin-dependent kinase inhibitor protein CIP1 (p21) that mediates cell cycle arrest, displayed a sharp increase in transcripts at 6 h while maintaining some degree of induction at 24 h time as well. Additionally, as could be expected from the EGF-like signalling described earlier for OA, the endogenous mRNA levels of the *EGF* gene dropped abruptly with the treatment, slightly bouncing back over control levels after 24 h.

In a different fashion, the mRNA levels of *SNAI2*, a gene encoding for a zinc finger transcription factor related to the modulation of the migratory status, just showed significant induction at 24 h time. Similarly, the *ITGB6* and *PAX* genes, which protein products drive the interaction of the cell surfaces and cytoskeleton with the ECM, exhibited some degree of induction appreciable only after long-term exposure. Furthermore, the expression levels of the MMP9 gene, which encodes for a zinc-metalloproteinase involved in ECM remodelling and wound healing [30, 31], followed a similar pattern. Noteworthy, Forkhead Box O1 (*FOXO1*) gene expression clearly increases at 24 h suggesting the transition to a wounding status of MDA-MB-231[32].

Taken together, these data reveal the ability of OA to alter the expression pattern of different genes related to cell migration and wound healing at several levels, in MDA-MB-231 cells.

### Oleanolic acid effects are mediated through EGF receptor activation

**Fig 7:**
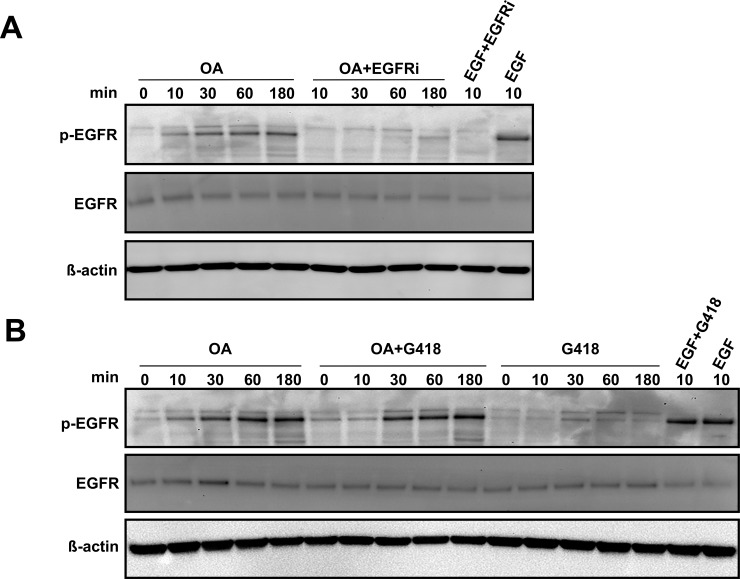
Oleanolic acid promotes direct EGFR activation in MDA-MB-231 cells. (A) The phosphorylation status of EGFR was assessed by Western Blot at the indicated times in the presence or absence of PD153035, EGF Receptor Inhibitor [EGFRi]. (B) The phosphorylation status of EGFR was assessed by Western Blot at the indicated times in the presence or absence of the ribosome inhibitor G418 (geneticin). Representative images of at least three independent experiment are shown.

In an effort to sort out the implication of EGFR in the observed effects, we decided to analyse its activation upon OA treatment. On ligand binding, EGFR undergoes a transition from monomeric form to an active dimer, triggering crossed auto-phosphorylation of tyrosine (Y) residues 992, 1068 and 1086 at the C-terminal domain, which result conformation changes that render the receptor activated [33]. Using an anti-phospho-EGFR antibody detecting phosphorylated Y1068 residue allowed us to observe increasing levels of activated receptor upon OA exposure in WB of MDA-MB-231 samples, achieving after 3 h intensity levels close to those reported for EGF control (Fig 7A). Interestingly, the same pattern was observed when this approach was carried out on protein extracts from MDA-MB-231 cells treated in the presence of the ribosome inhibitor geneticin (Fig 7B).

**Fig 8:**
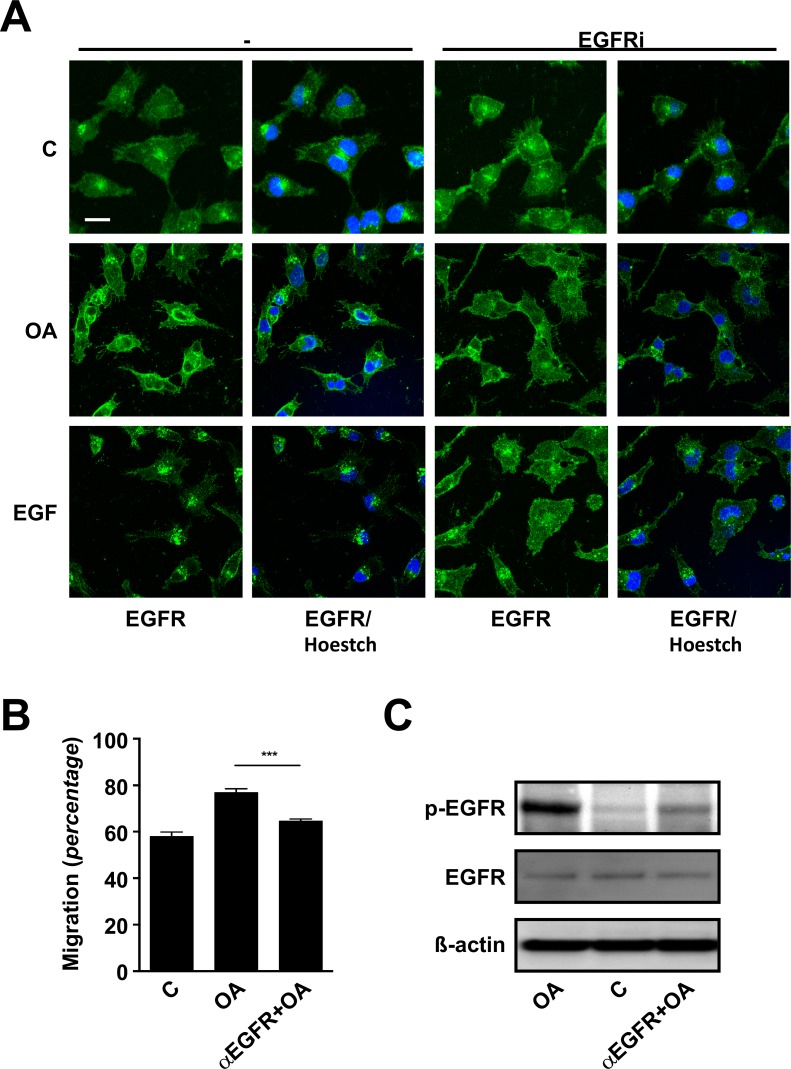
Oleanolic acid triggers subsequent internalization of active-EGFR in MDA-MB-231 cells. (A) The cellular distribution of EGFR after indicated treatments, in the presence or absence of PD153035, EGF Receptor Inhibitor [EGFRi], was assessed by means of confocal microscopy. Scale Bar 10 μm. (B) Wound healing scratch assay was performed on confluent MDA-MB-231 cells in the presence or absence of blocking EGFR antibody (40 μg/ml) and migration was determined. Shown data is average of three independent experiments. *p<0.05, **p<0.005, ***p<0.001 and ****p<0.0001. (C) The phosphorylation status of EGFR was assessed by Western Blot at the indicated times in the presence or absence of EGFR blocking antibody (40 μg/ml). Representative images of at least three independent experiment are shown.

EGFR physiology is known to be tightly regulated, including receptor internalization and both degradation and recycling mechanisms [34, 35]. Studying the receptor dynamics by means of immunocytochemistry, we found that after 1 h OA treatment triggers a delayed rather mild condensation of the fluorescence signal compared with the effects achieved by EGF just after 10 min. Worth noting, the effects of both EGF and OA on the EGFR dynamics were apparently abrogated when cells were stimulated in the presence of EGFRi (Fig 8A). Finally, after the previous observations, we decided to analyse migration on cells exposed to OA in the presence of a blocking anti-EGFR antibody [36]. Strikingly, we found that the presence of the blocking antibody hampered any improvement of cell migration by OA both in the case of Mv1Lu (data not shown) and MDA-MB-231 cells (Fig 8B). Indeed, when we assessed EGFR phosphorylation on samples subjected to same treatments, we detected a strong phosphorylation blockage in the case of samples pre-treated with anti-EGFR antibody and then stimulated with OA, in contrast to OA stimulated samples (Fig 8C).

Together, these results provide good evidence on the involvement of EGFR in the effects observed upon the treatment with OA on cell migration. Moreover, our data show that EGFR activation develops early after OA treatment, strongly suggesting the occurrence of a direct stimulation of the receptor.

## Discussion

In this paper, we pursued to disclose the molecular mechanisms underlying the positive effects of OA in wound healing from the cellular migration context. Studying its effects on two different cell lines with epithelial linage, we observed that OA clearly enhanced cell migration for *in vitro* scratch closure. This effect correlated with the activation of several proteins critical for cell migration to happen, as c-Jun phosphorylation and ERK1,2 phosphorylation. Moreover, gene expression analysis performed in the human cell line showed an expression profile accordant with the activation of a pro-migratory status and the ability for ECM remodelling, observations altogether consistent with the observed effects of OA on wound healing. Finally, we found that those effects correspond with the activation of EFGR, whose interference by means of immunological blockage or pharmacological inhibition prevents any enhancement of migration by OA.

The primary function of the skin is to serve as a protective barrier against the environment [2]. After disruption, wound healing progresses as a dynamic and interactive process involving soluble mediators, blood cells, ECM and parenchymal cells to reconstitute this barrier’s properties [2]. However, alterations of this process related to age or illness affect an increasing large number of patients suffering from chronic non-healing ulcers that eventually complicate into infection or necrosis [37]. This conundrum urges for the development of alternative therapies providing healing acceleration and reduced wound-related complications, which lately bolstered the search for cicatrizing chemicals derived from botanical sources used in traditional medicine [38]. Flavonoids have been identified as a major group of active compounds present in plants which have desired properties upon health and medical treatment [9]. Among them, pentacyclic triterpenes have been successfully used to treat experimental wound-healing in humans, outreaching wound closure and aesthetic outcome performance when compared to common clinical approaches [12]. Precisely among pentacyclic triterpenes, OA has been labelled in the past with explicit cicatrising properties, as increasing tensile strength of experimental wounds in mice [11].

During the formation of granulation tissue in a dermal wound, there is a general release of peptide growth factors which stimulate fibroblasts to migrate into the wound site and proliferate, in order to reconstitute the various connective tissue components [6]. Fibroblast migration to and proliferation within the wound site are prerequisite for wound granulation, since they participate in the removal of the fibrin clot and the construction of a “substrate layer” to facilitate other cell types actions [6]. Consequently, the modulation of fibroblast activity by peptide growth factors is reported as responsible for improved wound healing [6]. Interestingly fibroblast, which are mesenchymal-derived cells, had been shown to migrate in response to OA leading to enhanced wound closure [14]. In our case, working with *in* vitro scratch assays, we found that OA significantly enhances cell migration even tough used at low concentrations. As already mentioned, wound closure is both contributed by cell migration and proliferation, thus, it was not surprising when we noted that the observed properties of OA were less evident when we provided serum supplementation to cells. In this situation, trophic factors present in the medium are not only promoting higher migration capacity but also boost proliferative activity, altogether eventually masking OA’s contribution to the scratch closure. Hence, we performed a series of experiments designed to ponder the relative importance of OA’s effects on migration related to cell proliferation. MMC is a chemotherapeutic drug known to promote DNA crosslinking and thus halt cell division [39]. Remarkably, after removing proliferation’s contribution to migration by MMC exposure of Mv1Lu cells, OA treatment still retained significant migration enhancement properties, thus supporting the notion that some of its observed properties would be detached from proliferation and thus contribute separately to the healing process. In this sense, using serum deprived conditions, a threshold for OA’s concentration was observed, as doses higher than 5 μM or 10 μM for Mv1Lu and MDA-MB-231 cells, respectively, showed to be detrimental for the migration outcome. Nevertheless, high concentrations of OA had been previously reported to interfere with proliferation in NIH/3T3 fibroblasts [14]. Moreover, despite confirming OA’s detrimental effects on proliferation when serum supplemented conditions are used for both Mv1Lu and MDA-MB-231 cell lines, these effects seem to be short lived, as no alteration in cell cycle distribution was recorded at long term. Whether this observation is the consequence of a transitory arrest in cell cycle, perhaps due to the degradation of OA in the media or its metabolization by cells, it will require additional research to be assessed properly. Anyhow, even though a slight decrease in proliferative activity could be recorded by short-term cell culture PHH3 immune-labelling, it is clear that those effects do not jeopardize the properties of OA, which both at conditions where proliferation was impaired or remained untouched, displayed a net contribution to cell migration in these cells.

It is well stablished that MAP kinases family members participate in the promotion of cell motility, as growth factors and integrins can activate signalling events leading to MAP kinase activity and the induction of cell migration [40–44]. Previous reports had shown the ability of OA to affect MAP kinases, reducing migration of glioma cells [45]. Here, we also show the key involvement of MAP kinases, but in this case in the promotion of migration by OA on epithelial cells. Activated JNK has been shown to play a critical role in the migration of fibroblasts in wound healing assays, while the exposure to the JNKi has been shown to negatively affect the migration of numerous cell types [43, 46]. In our case, for both Mv1Lu and MDA-MB-231 cell lines, OA treatment triggered the phosphorylation of JNK1/2, which activation brought increased detection of phosphorylated c-Jun. This represent an excellent readout for the migratory status of epithelial cells, since keratinocytes lacking active c-Jun are unable to migrate or elongate properly in culture at the border of wound healing scratch assays [47]. Moreover, JNKi showed to effectively impair the effects of OA on migration, although some improvement still remained. In the same line, it has been shown that direct suppression of ERK1/2 or inhibition of its phosphorylation by inhibiting MEK1 activity prevents cell migration in human keratinocyte [40]. Again in our case, the activation of MEK1 seems to be also required for migration, since ERK1/2 phosphorylation its detected upon treatment and the use of MEKi affected migration in a similar degree to JNKi. While this further demonstrates the involvement of both MAP kinase pathways in the OA properties on migration, their individual contributions seem to be necessary but not sufficient. Interestingly, 2',6 Dichloro-7-methoxyisoflavone (DCMF), a synthetic flavonoid, has been recently shown to promote human keratinocyte migration by activating Src/FAK signalling, which renders the activation of ERK, AKT and p38 signalling pathways [48]. p38, also a MAP kinase, is considered a key player in the intracellular signalling events related to environmental stress and immune insult. However, in our hands, the stimulation of migration by OA was not prevented by p38 inhibitor, an interesting result that may lay in the distinct chemical nature of both flavonoids, OA being a triterpene versus DCMF being an isoflavone. While this difference may have an impact on the potential clinical properties of DCMF or OA, it is clear that the enrolment of MAP kinases family members in the promotion of cell migration by flavonoids plays a capital role.

While it is known that activation of the MAP kinase pathways leads to transcriptional control of genes important for cell proliferation and differentiation [49], on the other hand, it has been also suggested that MAP kinase activation can drive direct activation of the intracellular motility machinery [40]. We aimed to clarify whether or not OA’s observed stimulation of the JNK and ERK MAP kinases pathways has an effect on the gene expression profile of migrating MDA-MB-231 cells, as our WB data showed that OA stimulated not only the phosphorylation of c-Jun but also increased its protein levels. Indeed, one of the effects of OA was the lasting increase of *c-JUN* gene expression. It has been shown that up-regulation of c-Jun is detected in cells at the leading edge during wound healing [47, 50]. Moreover, c-Jun has been found to be crucial in conditions where the proliferation and motility of the epithelium are quickly changed [43]. In a parallel way, one of the most interesting effects of OA is the sharp induction of the *CDKN1A* gene, which was also corroborated by WB (S6 Fig). *CDKN1A* encodes for the protein p21, which promotes cell cycle arrest in response to many stimuli [51]. Hence, the counterbalance provided by p21 may be a suitable explanation to the lack of pro-proliferative perhaps slight anti-proliferative effects exerted by OA. In one way or another, the effect of OA in the expression of these genes is evident and eventually decrease with time, an observation which coherent with the lack of proliferative alteration previously discussed.

OA also induces the expression of genes involved in the regulation of cell migration. Interestingly, *SNAI2* and *FOXO1*, which are bot transcription factors, display a similar expression profile. *SNAI2* belongs to the Snail family of zinc finger transcription factors. It plays an important role in Epithelial to Mesenchymal Transition (EMT) during wound re-epithelialisation and its expression has been shown elevated in keratinocytes at the margins of healing wounds in mouse and human *in vivo* and *in vitro* models [52, 53]. Similarly, *FOXO1* has been recently postulated to be necessary for keratinocytes to undergo EMT and acquire a wound-healing phenotype. Curiously, those observations involved the up-regulation of Transforming Growth Factor ß1 (TGF-ß1), commonly referred as to promoting cell cycle arrest [32, 54]. In either way, during wound healing, this transcription factor balance is responsible for different cell types fate. For instance, keratinocytes at the leading edge migrate over injured dermis, whereas only keratinocytes behind the leading edge proliferate and mature [22, 55–57]. Consequently, we believe that the change in expression of these transcription factor genes, along with *c-JUN*, in response to OA, is consistent with the observed effects on cell migration and proliferation, in relation to the activation of an EMT condition.

In respect to genes involved directly in migration events and EMT, JNK-c-jun signalling pathway is known to lead induction of several downstream target genes, one of which is the type 1 plasminogen activator inhibitor (*PAI-1*) [28, 58]. During wound healing, elevated levels of PAI-1 protect ECM proteins from proteolytic degradation, which facilitates re-epithelialization of the wound bed by promoting successful cellular attachment [29, 59, 60]. In our model, the clear induction of *PAI1* by OA, might be related to the preservation of ECM elements that ensure successful cell attachment during cell migration. In that sense, the rest genes studied in this paper depicting a late response to OA treatment, are also known to directly intervene in migration and tissue remodelling. Precisely, the expression of integrin subunit beta 6 (*ITGB6*), a cell-surface adhesion receptor that mediate cell-ECM interactions, had been related to enhanced cell migration in the context of human neoplasia and wound healing [61]. Morevover, the matrix metallo-proteinase 9 (*MMP9*) gene, which main function is to cleavage denatured collagens of all types, is known be induced in cells at the front of the migrating epithelial sheet as it begins to resurface the wound bed following injury [62]. Finally, Paxillin (*PXN*) is a key scaffold protein in the assembly and function of cell focal adhesions [63]. In this case, the slight mRNA induction observed could not be verified on WB, as protein levels remained steady after OA treatment (S6 Fig). However, we find this fact would be in agreement with the known turnover that focal adhesions experience during cellular migration [64]. Altogether, we believe that the modulation of genes discussed above, support the conformation of a molecular framework necessary for cytoskeleton reconfiguration, ECM remodelling, and hence cell migration, in MDA-MB-231 cells treated with OA, in line with the observed effects in scratch assays for both Mv1Lu and MDA-MB-231 cell lines.

Ursolic acid is other triterpenoid compound with similar structure to OA, which has been shown to have anti-proliferative and pro-apoptotic effects on both human and mouse colon cancer models [65, 66]. Those effects appear to be driven by the direct interaction of Ursolic acid with the EGFR, ultimately leading to the inhibition of the EGF/MAP kinases signalling pathway [65, 66]. According to our data, these observations would support a potential agonistic activity of OA on EGFR. Interestingly, in our case for both Mv1Lu and MDA-MB-231 cell lines, EGF and OA co-treatment never significantly outreached the migration displayed by cells treated just with EGF, suggesting that the observed pro-migratory effects of OA could be channelled through the same system. Intriguingly, the use of EGFRi [67] completely abrogated any effects of OA on cell migration, while the combination of both MEKi and JNKi performed similarly to EGFRi. As this pointed to EGFR as a common up-stream regulator for both JNK1/2 and ERK1/2 pathways in the case of OA stimulation, we looked deeper into EGFR activation. Surprisingly, OA treatment not only resulted in a steady receptor activation, but we also found a noticeable drop in the expression of the *EGF* gene, which meets the well stablished existence of negative feedback mechanisms for EGF signalling [68]. Moreover, OA promoted the internalization of EGFR from the cell membrane, in line with what has been described in the case of stimulation with EGF, as well as other EGFR ligands [34, 35]. Indeed, while inhibiting translation with geneticin did not alter the activation of EGFR, we found that the use of a specific EGFR blocking antibody [36] successfully prevented OA effects. Albeit these observations suggest that this mechanism will be independent of *de novo* synthesized secondary messengers, neither a direct agonistic activity of OA nor the indirect activation of EGFR by ligands released upon OA stimulation could be discarded. Nevertheless, in the light of our results, we favour the possibility of OA for tweaking ligand-receptor interactions thus enhancing autocrine EGFR-signalling loops, as described in polarized epithelial cells [69], which would better explain OA observed effects on migration as well as on proliferation.

Upon dermal barrier disruption tissue constituents must transient from previous homeostasis (resting or programmed proliferation), to new phenotypes that facilitate tissue reparation. In this paper, we provide molecular evidence on the properties of OA for the rapid enhancement of traits desired in epithelial cells for wound-healing, while regulating a proper balance between proliferation and migration. Surprisingly, those observations are made on epithelial cells from distant origins and species, perhaps suggesting the universality of the mechanisms involved in such fundamental aspect key for wound-healing outcome. Furthermore, we demonstrate that the treatment triggers molecular signalling pathways involving EGFR and leading to the development of a gene expression profile consistent with observed migration and the acquisition of tissue remodelling abilities. In conclusion, we demonstrate that the effects described for some traditional medicine preparations from plant extract used in wound healing, do have a molecular explanation in the face of OA and its interactions, knowledge which may open opportunity for future research and innovation in the area of wound healing and regeneration.

## Supporting information

S1 Fig Oleanolic acid induces motility in Mv1Lu cells at low doses in serum deprived conditions. Cell migration is represented as variation of the area differences between treatments and control samples for each assay (percentage). (A) Increasing OA concentrations were administered with medium containing 10% serum. Notice that OA effects are restricted to higher doses. (B) Increasing OA concentrations were administered in serum deprived conditions. Low and intermediate doses enhanced cell migration. In all cases, plots are representative of three independent experiments. *p<0.05, **p<0.005, ***p<0.001 and ****p<0.0001.
(TIF)

S2 Fig Oleanolic acid stimulates Mv1Lu migration. Representative pictures of scratch wound assays after 18 h of incubation in serum-free medium in the conditions indicated. Inhibitors nomenclature: SP600125, JNK inhibitor [JNKi]; PD98059, MEK1 inhibitor [MEKi] or PD153035, EGF Receptor Inhibitor [EGFRi]. Scale Bar 200 μm.
(TIF)

S3 Fig Oleanolic acid reduces Mv1Lu cells phospho-Histone H3 immuno-labeling after 24 h. Effects of OA on Mv1Lu proliferation were assessed by phospho-Histone H3 immuno-labeling and the average dividing cells number was quantified by determining the number of positive cells per field. (A) Immuno-labeling of cells cultured in serum supplemented conditions. A representative picture is shown. (B) Average positive cells, serum supplemented conditions. (C) Immuno-labeling of cells cultured in serum deprived conditions. A representative picture is shown. (D) Average positive cells, serum deprived conditions. Scale Bar 50 μm. *p<0.05, **p<0.005, ***p<0.001 and ****p<0.0001.
(TIF)

S4 Fig Oleanolic acid displays marginal effects on MDA-MB-231 cells migration while reduces cell proliferation in serum supplemented conditions. (A) Increasing OA concentrations were administered with medium containing 10% serum. (B) Increasing OA concentrations were administered in serum deprived conditions. (C) Phospho-Histone H3 immuno-labeling of MDA-MB-231 cells exposed to OA for 24 h in serum deprived conditions. (D) Average positive cells number was quantified by determining the number of positive cells per field. (E) Phospho-Histone H3 immuno-labeling of MDA-MB-231 cells exposed to OA for 24 h in serum supplemented conditions. (F) Average positive cells number was quantified by determining the number of positive cells per field. Representative pictures are shown. Scale Bar 50 μm *p<0.05, **p<0.005, ***p<0.001 and ****p<0.0001.
(TIF)

S5 Fig Oleanolic acid stimulates MDA-MB-231 migration. Representative pictures of scratch wound assays after 19 h of incubation in serum-free medium in the conditions indicated. Inhibitors nomenclature: SP600125, JNK inhibitor [JNKi]; PD98059, MEK1 inhibitor [MEKi] or PD153035, EGF Receptor Inhibitor [EGFRi]. Scale Bar 200 μm.
(TIF)

S6 Fig Effects of Oleanolic acid on protein expression. Levels of *CDKN1A* gene protein product (p21) or *PAX* gene protein product (Paxillin) were assessed by Western Blot along with ß-actin as loading control. A representative image of at least three independent experiment is shown.
(TIF)
