# Supplementary figures and images for: Oleanolic acid induces migration in Mv1Lu and MDA-MB-231 epithelial cells involving EGF receptor and MAP kinases activation
Source: PLoS One. 2017 Feb 23;12(2):e0172574. doi: 10.1371/journal.pone.0172574 (PMC5323077; doi:10.1371/journal.pone.0172574)

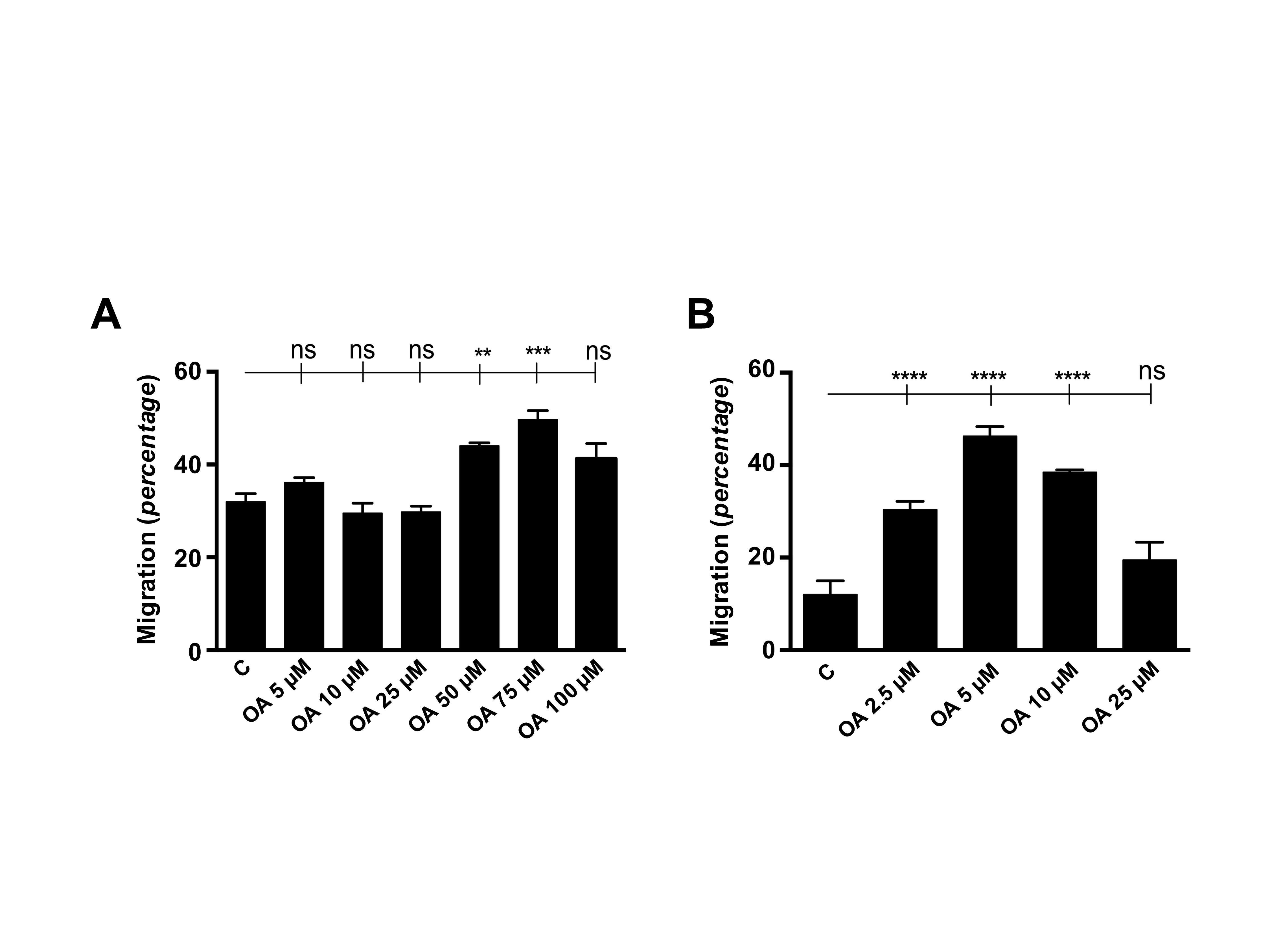

Supplement: S1 Fig — Cell migration is represented as variation of the area differences between treatments and control samples for each assay (percentage). (A) Increasing OA concentrations were administered with medium containing 10% serum. Notice that OA effects are restricted to higher doses. (B) Increasing OA concentrations were administered in serum deprived conditions. Low and intermediate doses enhanced cell migration. In all cases, plots are representative of three independent experiments. *p<0.05, **p<0.005, ***p<0.001 and ****p<0.0001. (TIF) [file pone.0172574.s001.tif]

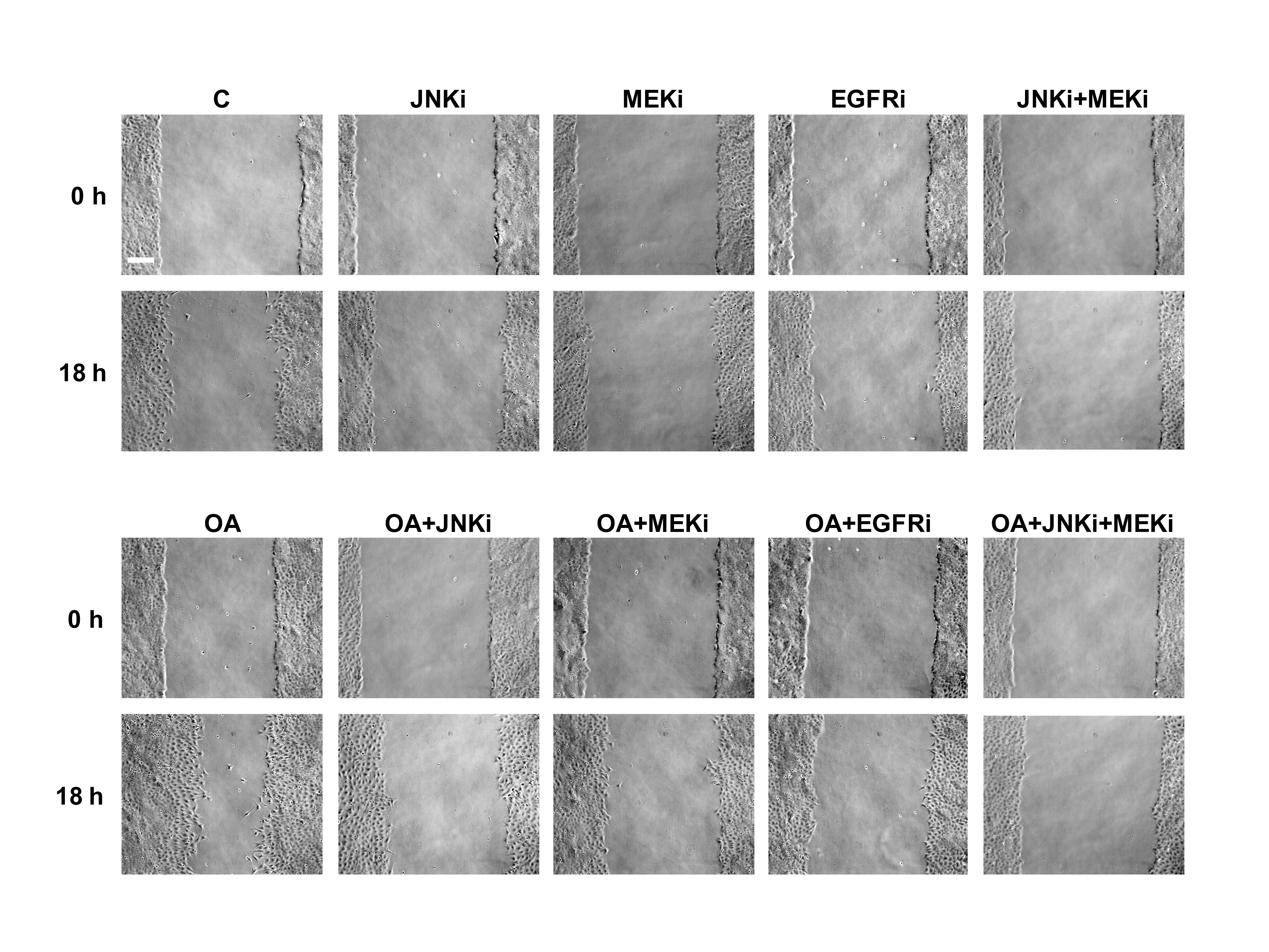

Supplement: S2 Fig — Representative pictures of scratch wound assays after 18 h of incubation in serum-free medium in the conditions indicated. Inhibitors nomenclature: SP600125, JNK inhibitor [JNKi]; PD98059, MEK1 inhibitor [MEKi] or PD153035, EGF Receptor Inhibitor [EGFRi]. Scale Bar 200 μm. (TIF) [file pone.0172574.s002.tif]

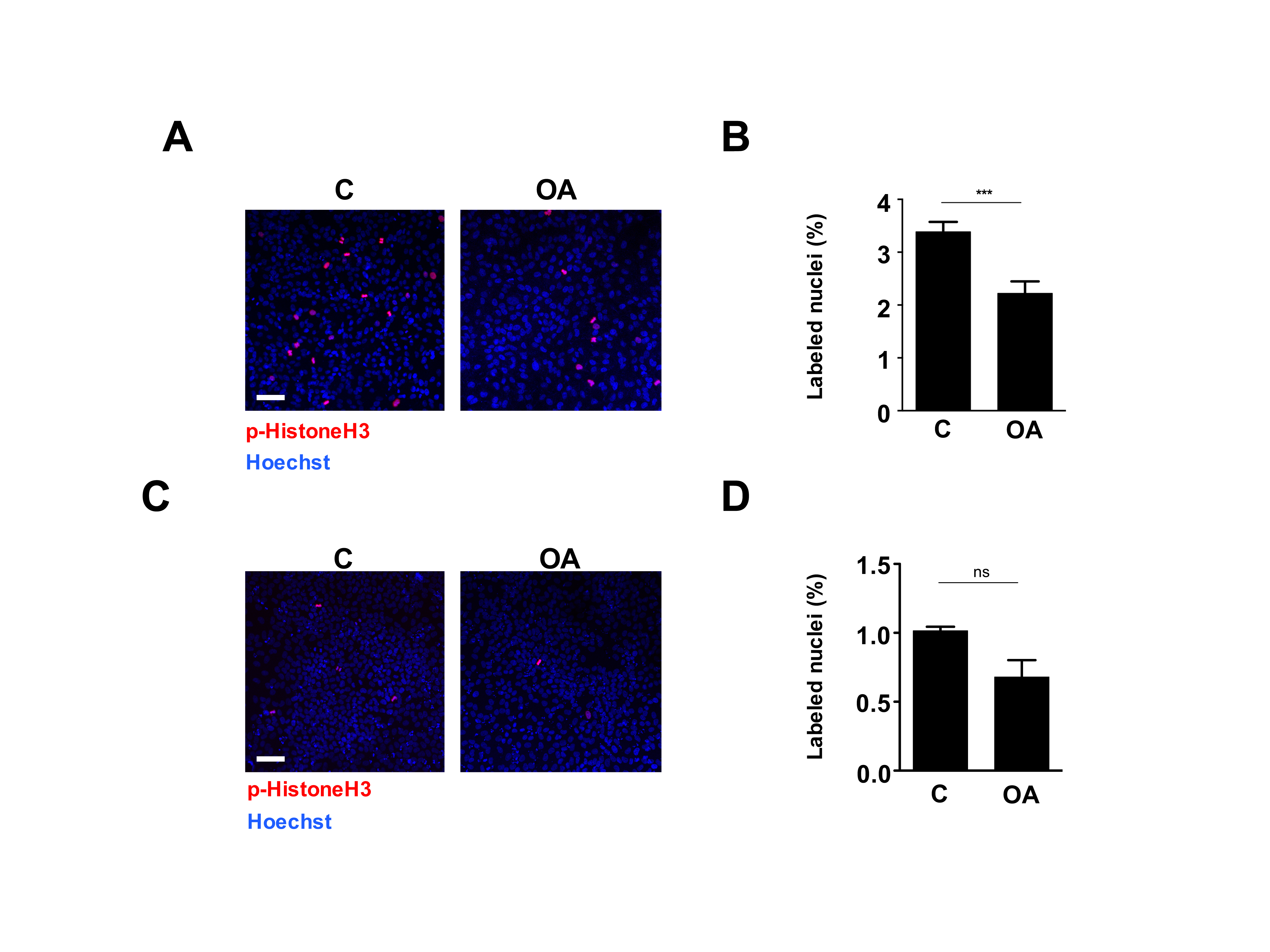

Supplement: S3 Fig — Effects of OA on Mv1Lu proliferation were assessed by phospho-Histone H3 immuno-labeling and the average dividing cells number was quantified by determining the number of positive cells per field. (A) Immuno-labeling of cells cultured in serum supplemented conditions. A representative picture is shown. (B) Average positive cells, serum supplemented conditions. (C) Immuno-labeling of cells cultured in serum deprived conditions. A representative picture is shown. (D) Average positive cells, serum deprived conditions. Scale Bar 50 μm. *p<0.05, **p<0.005, ***p<0.001 and ****p<0.0001. (TIF) [file pone.0172574.s003.tif]

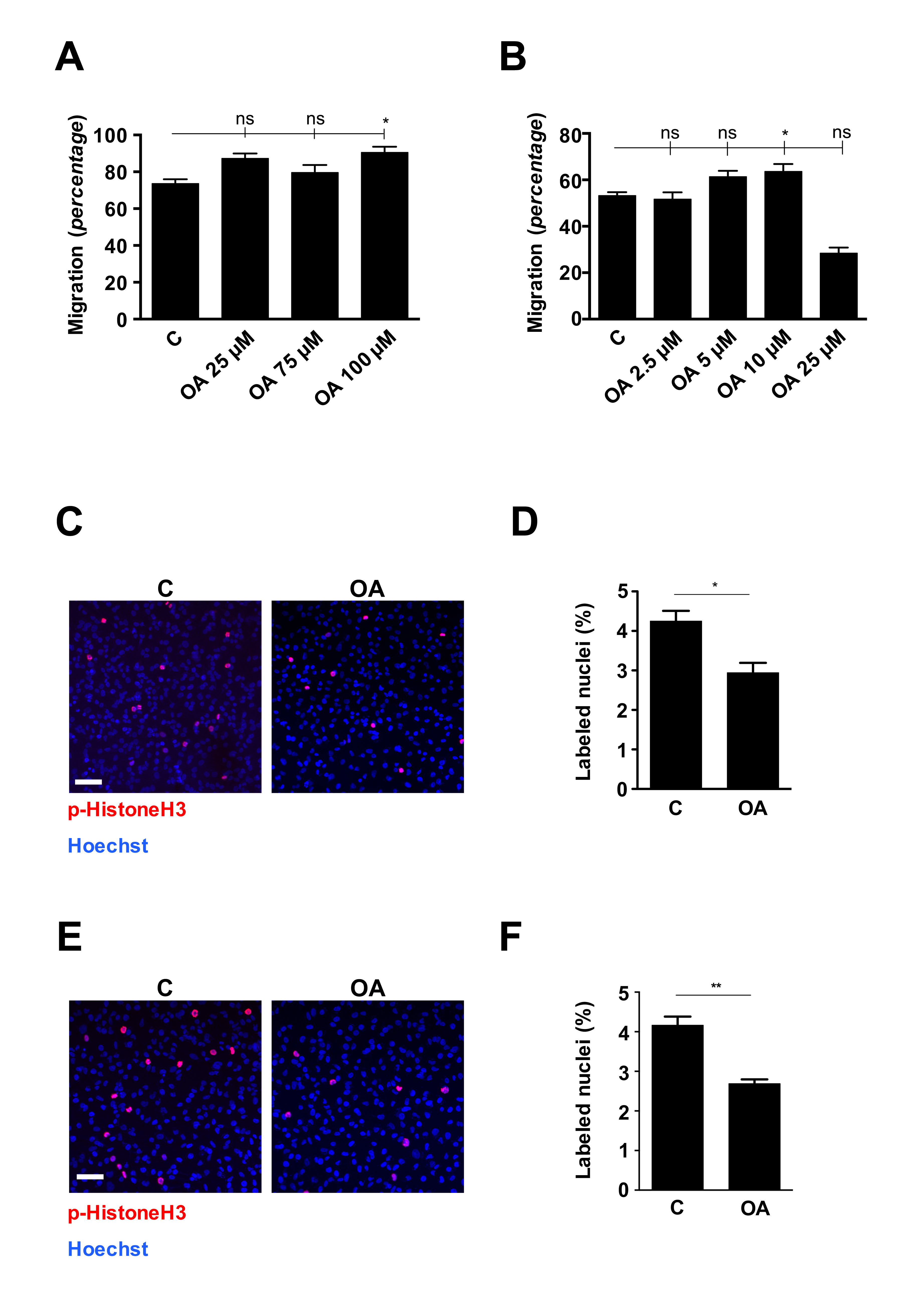

Supplement: S4 Fig — (A) Increasing OA concentrations were administered with medium containing 10% serum. (B) Increasing OA concentrations were administered in serum deprived conditions. (C) Phospho-Histone H3 immuno-labeling of MDA-MB-231 cells exposed to OA for 24 h in serum deprived conditions. (D) Average positive cells number was quantified by determining the number of positive cells per field. (E) Phospho-Histone H3 immuno-labeling of MDA-MB-231 cells exposed to OA for 24 h in serum supplemented conditions. (F) Average positive cells number was quantified by determining the number of positive cells per field. Representative pictures are shown. Scale Bar 50 μm *p<0.05, **p<0.005, ***p<0.001 and ****p<0.0001. (TIF) [file pone.0172574.s004.tif]

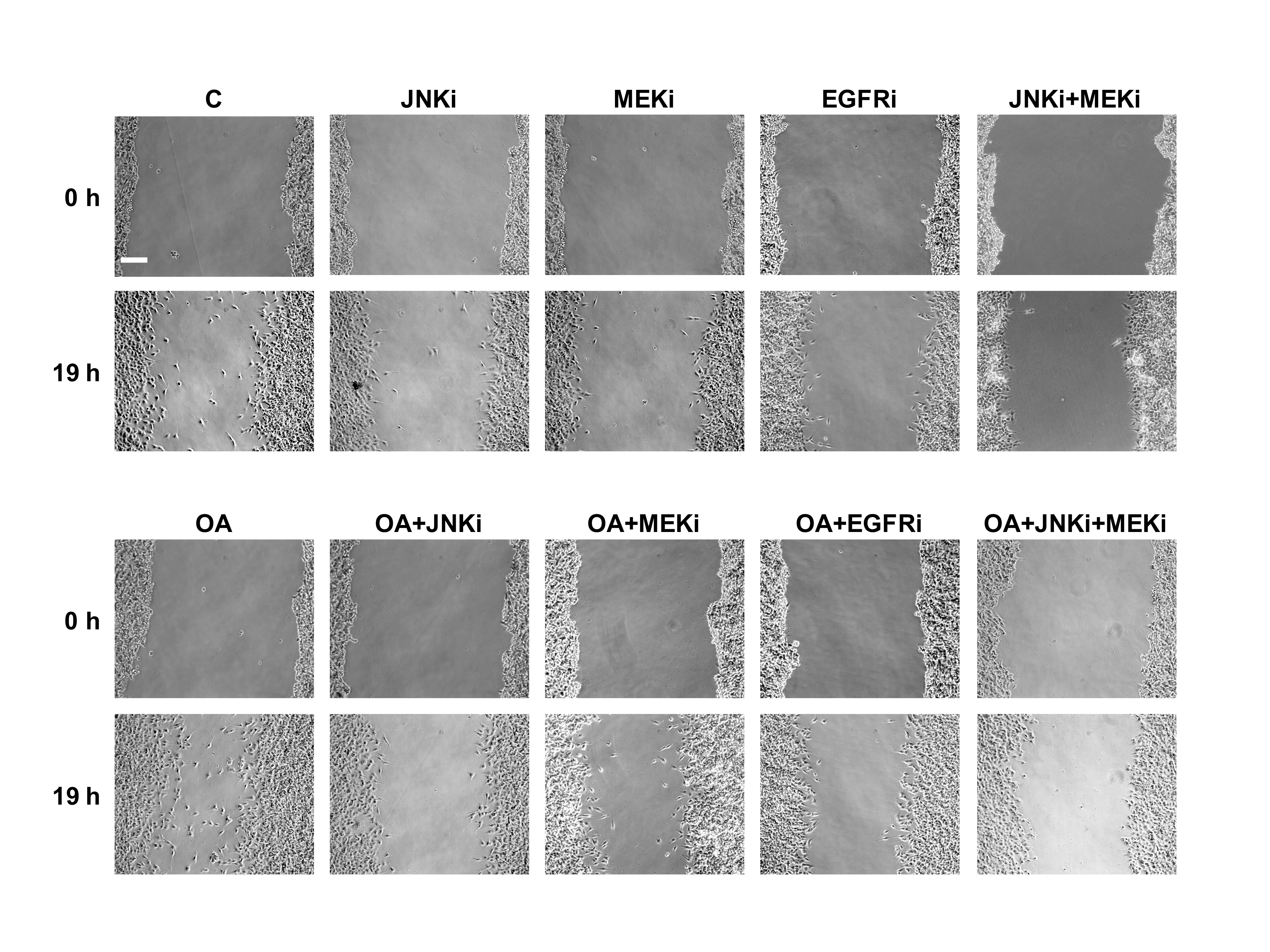

Supplement: S5 Fig — Representative pictures of scratch wound assays after 19 h of incubation in serum-free medium in the conditions indicated. Inhibitors nomenclature: SP600125, JNK inhibitor [JNKi]; PD98059, MEK1 inhibitor [MEKi] or PD153035, EGF Receptor Inhibitor [EGFRi]. Scale Bar 200 μm. (TIF) [file pone.0172574.s005.tif]

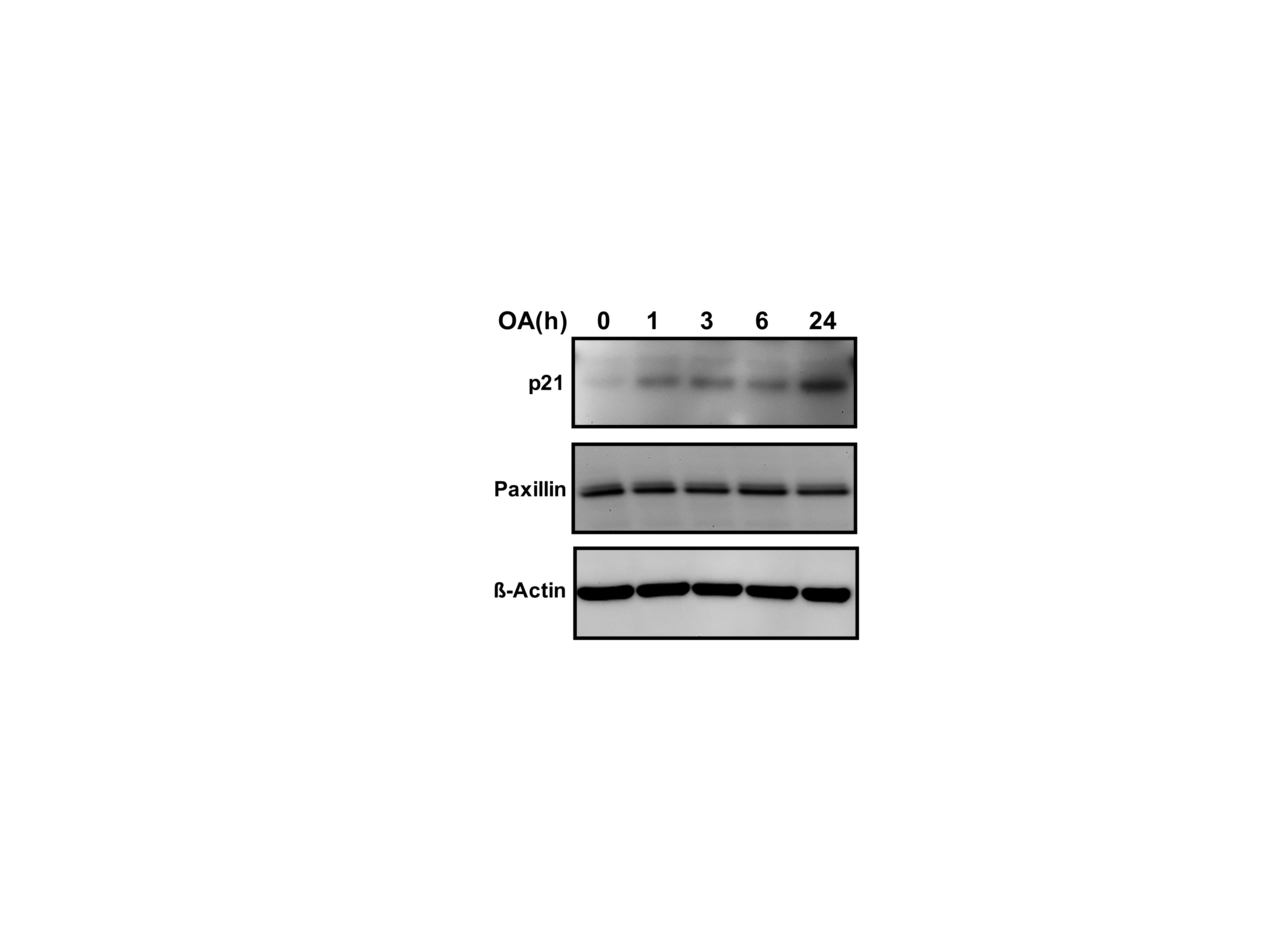

Supplement: S6 Fig — Levels of CDKN1A gene protein product (p21) or PAX gene protein product (Paxillin) were assessed by Western Blot along with ß-actin as loading control. A representative image of at least three independent experiment is shown. (TIF) [file pone.0172574.s006.tif]
